# The effect of the egg-predator *Carcinonemertes conanobrieni* on the reproductive performance of the Caribbean spiny lobster *Panulirus argus*

**DOI:** 10.1186/s40850-023-00165-w

**Published:** 2023-06-26

**Authors:** Amanda Berben, Natalie C. Stephens, Jaime Gonzalez-Cueto, Yulibeth Velasquez, Sigmer Quiroga, María Teresa González, J. Antonio Baeza

**Affiliations:** 1 Universidad del Magdalena, Santa Marta, Colombia; 2 Department of Biological Sciences, Clemson University, 132 Long Hall, Clemson, SC 29634 USA; 3 Facultad de Ciencias del Mar Y Recursos Biológicos, Instituto de Ciencias Naturales “Alexander Von Humboldt”, Universidad de Antofagasta, Angamos, 601 Antofagasta, Chile; 4 Smithsonian Marine Station at Fort Pierce, 701 Seaway Drive, Fort Pierce, FL 34949 USA; 5 Departamento de Biología Marina, Facultad de Ciencias del Mar, Universidad Católica del Norte, Larrondo 1281, Coquimbo, Chile

**Keywords:** Ribbon worms, Disease, Reproductive performance, Epidemiology, Pathogen

## Abstract

**Background:**

The Caribbean spiny lobster *Panulirus argus* is heavily fished throughout its Greater Caribbean and Gulf of Mexico distribution, suggesting a heightened susceptibility to a fisheries collapse. In 2017, a nemertean worm, *Carcinonemertes conanobrieni* was described from ovigerous females of *P. argus* in Florida, USA. A year later, the presence of the same egg predator was recorded along the southern Caribbean coast (Colombia). The effect of this egg predator on the reproductive performance, including fecundity, embryo mortality, and reproductive output, of its host is unknown. This study tested whether *C. conanobrieni* affects embryo mortality, fecundity, and reproductive output in brooding females of *P. argus*.

**Results:**

Artisan fishers caught 90 ovigerous lobsters near Pueblo Viejo, Magdalena, Colombia. Each ovigerous female was examined for the presence/absence of the egg predator. Lobster egg mortality (%), fecundity (nº eggs female^−1^), and reproductive output (%) were estimated. Prevalence of *C. conanobrieni* in the studied population was 87.78%. The mean intensity of *C. conanobrieni* (all life stages) in the population was 11.68 (± 1.98) egg predators per brood mass sample. Infected females brooding late-stage embryos exhibited lower fecundity, lower reproductive performance values, and higher embryo mortality compared to infected females brooding early-stage embryos. Embryo stage and worm infection level negatively impacted fecundity and reproductive output. Worm infection level and the number of adult nemertean worms also negatively affected embryo mortality.

**Conclusions:**

These results demonstrate an adverse effect of *C. conanobrieni* on the reproductive performance of *P. argus*. The interactive impact of this egg predator, natural stressors, and anthropogenic stressors on individual *P. argus* reproductive performance could facilitate losses at large-scale fisheries levels.

**Supplementary Information:**

The online version contains supplementary material available at 10.1186/s40850-023-00165-w.

## Background

**Fig. 1:**
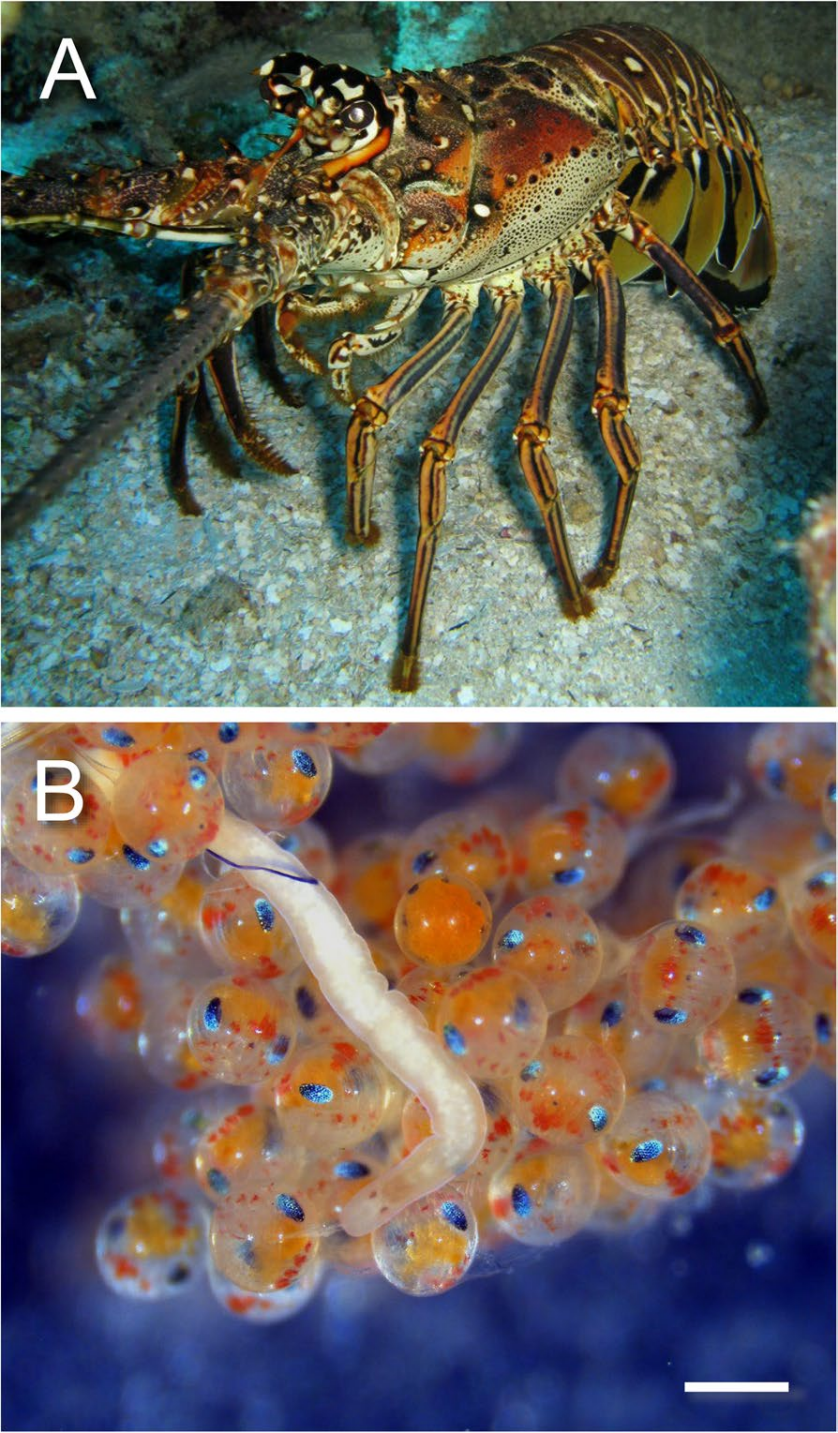
The Caribbean spiny lobster *Panulirus argus *(**A**) and its egg-predator worm *Carcinonemertes conanobrieni* (**B**)

The Caribbean spiny lobster *Panulirus argus* (Latreille 1804) is the target of one of the most valuable fisheries in the Greater Caribbean region, including the Gulf of Mexico, making it more susceptible to a fisheries collapse. *Panulirus argus* landings support a billion-dollar industrial fleet and artisan fishery across both developed and developing countries throughout its entire distribution, and this species represents a commodity that sells for higher market prices compared to other fished marine resources (between 5–8 USD/Kg) [1, 2] (Fig. 1A). Importantly, many moderate- to low-income level coastal communities in developing countries such as Honduras, Belize, and Guatemala rely either partially or almost entirely on this resource [3]. In Colombia, about 195 tons of *P. argus* were extracted in 2019 alone, which contributes to its status as a vulnerable species in this country [4, 5]. Local and global stressors, including pollution, habitat deterioration, and habitat destruction, have been hypothesized to contribute to declines in *P. argus* recruitment and landings in the greater Caribbean Sea [6–8]. Furthermore, diseases including but not limited to microsporidiosis, *Panulirus argus* Virus 1 (PaV1), tail fan necrosis, and trematode parasites found in this species may cause further complications for *P. argus* population health [9]. It is critical to understand the impact of the aforementioned disease agents including emerging pathogens, such as nemertean worms, on individual- and population-level reproductive parameters of *P*. *argus* in order to advance management and conservation policies in this lucrative fishery.

Limited data suggest that declines in female *P. argus* reproductive performance is attributable to a new nemertean egg predator belonging to the genus *Carcinonemertes* described from among the eggs of brooding female lobsters [10] (Fig. 1B). In 2017, *Carcinonemertes conanobrieni* was described from among the eggs brooded by female *P. argus* in the Florida Keys, USA [11]. A year later, the presence of the same worm was recorded in the Caribbean coast of Colombia, and most recently in the Eastern Caribbean Sea, specifically Saint Kitts and Nevis [12, 13]. Worms in the family Carcinonemertidae, including the genus *Carcinonemertes*, are recognized as voracious egg-predators that infect a variety of decapod crustaceans [14, 15]. *Carcinonemertes* worms are responsible for the collapse of crustacean fisheries in the west coast of North America [16–19] given their negative effect on female reproductive performance [20–24].

The nemertean worm *Carcinonemertes conanobrieni* has been recorded with variable prevalence in female *P. argus* populations across different localities of the Caribbean [9, 10, 12, 13, 24]. While initially discovered in the Florida Keys, the growing detection of *C. conanobrieni* among the brood masses of female *P. argus* brings into question how long this worm has been on the host, undetected, in previous years. Moreover, researchers have hypothesized that this egg predator may be present across *P. argus’* entire Caribbean distribution [12, 13]. *Carcinonemertes conanobrieni* is a voracious egg predator, known to leave the embryos of its host partially or fully consumed [24], which suggests that this egg predator can cause significant embryo mortality in females of this spiny lobster species. Specifically, *Carcinonemertes* worms exhibit suctorial feeding where the worm punctures the egg membrane with its stylet and withdraws the contents of the egg until partially or fully consumed [11, 24]. In addition to egg consumption, the worm displays a variety of behaviors when present in female egg masses including encysting alongside host eggs, finding refuge within mucus sheaths, or free roaming among the lobster eggs [9, 24]. Since the discovery of *C. conanobrieni* in populations of *P. argus* on the Caribbean coast of Colombia [12], many concerns have arisen regarding the negative ecological and economic effects of this egg predator. Limited data indicate that reproductive performance of female Caribbean spiny lobsters is negatively impacted in the presence of this worm. Notably, declines in fecundity and reproductive output were observed in females carrying early and late-stage embryos when infected with *C. conanobrieni* [10, 24].

The aim of this study was to evaluate the effect of the nemertean egg-predator *C. conanobrieni* on the reproductive performance of female *P. argus* in the southern Caribbean Sea. For this study, we chose to describe the worm as an egg-predator instead of a parasite because of the disproportionate, negative impact the worm has on the embryos of brooding females. Specifically, we investigated *C. conanobrieni* prevalence, intensity of infection, and its contribution to embryo mortality on *P. argus* brood masses. Individual-level lobster reproductive performance was measured via reproductive output and fecundity in infected and healthy females. Redundancy analysis was used to identify the effects of *C. conanobrieni* on reproductive performance and to identify host traits that explain the variation in worm life stages present on the hosts’ brood mass. By understanding the effect of *C. conanobrieni* on *P. argus* reproduction, we will be able to establish a baseline estimation of infection in the Colombia *P. argus* fishery. This will allow us to translate the results of this study to other populations across *P. argus*’ entire Caribbean distribution when infected with *C. conanobrieni*.

## Results

### Egg predator prevalence, intensity, and egg mortality

*Carcinonemertes conanobrieni* prevalence and intensity was high in the 38 and 52 female *P. argus* carrying early (I and II) and late (III and IV) stage embryos [10] under their abdomen, respectively. The carapace length (CL) of the 90 studied ovigerous females’ ranged between 61.04 and 133.2 mm with an average (± standard deviation, SD) of 99.87 (± 13.90) mm CL. Prevalence of *C. conanobrieni* in brooding female *P. argus*, estimated as the presence/absence of either free-roaming worms or worms within a shaft, was 77.78%. We observed eleven lobsters in which we failed to detect worms; however, worm cysts and egg masses were present. If we consider lobsters with at least one nemertean life stage or egg mass as infected, prevalence of *C. conanobrieni* in brooding females of *P. argus* increases to 87.78%.

A total of 923 *C. conanobrieni* specimens (including all worm life stages: adults, juveniles, and egg masses) were found in studied *P. argus* egg masses. Egg predator intensity varied considerably, between one egg predator per egg mass in five females (two females carrying early eggs and three females carrying late eggs) ranging between 61.04 and 127.5 mm CL (average ± SD = 66.46 ± 26.31 mm) and 72 egg predators per sampled egg mass in one female of 89.5 mm CL that carried late eggs. The mean intensity of *C. conanobrieni* (all life stages) in the population was 11.68 (± 1.98) egg predators per egg mass sample. The intensity of *C. conanobrieni* non-adult forms (cysts and egg masses) ranged between one and 8 (average ± SD = 2.55 ± 2.12), and the intensity of *C. conanobrieni* adult worms exclusively varied between one and 16 (average ± SD = 6.8 ± 4.98).

Embryo mortality was not observed in any non-infected (*C. conanobrieni* absent) gravid female lobsters, suggesting a strong negative impact of this egg predator on the host. Embryo mortality, indicated by empty capsules and dead embryos due to infection by *C. conanobrieni*, for females carrying early embryos ranged between 0% and 43.81% of egg masses (average ± SD = 3.90% ± 9.36). Additionally, we observed that infected *P. argus* carrying late-stage embryos had mortality rates between 0% and 48.24% (average ± SD = 9.22% ± 10.20). Females with 0% embryo mortality lacked any *C. conanobrieni* life stages among the embryo subsamples examined.

### Lobster fecundity and reproductive output (RO)

**Fig. 2:**
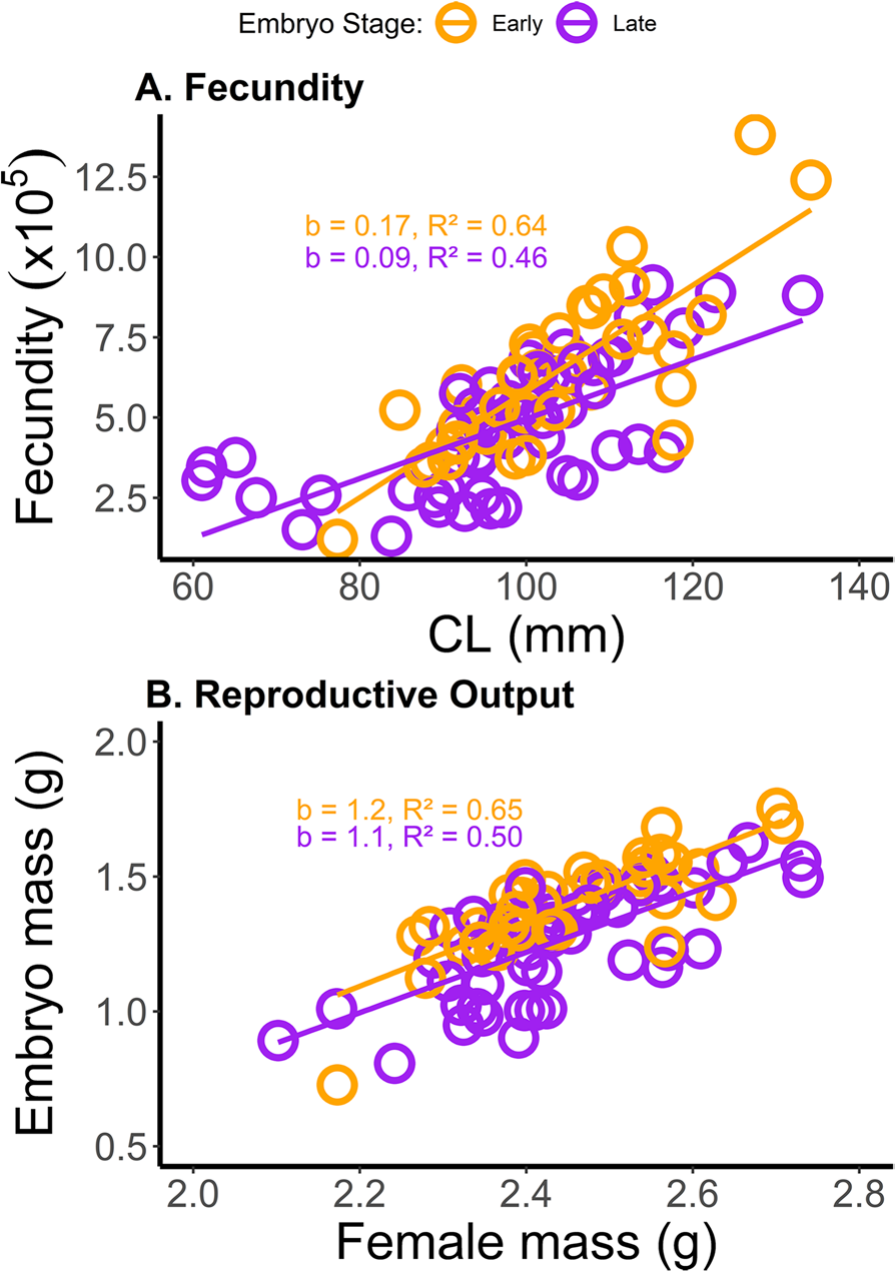
Reproductive performance of gravid females in *P. argus*. Reproductive performance of gravid females was significantly impacted by the infection of *C. conanobrieni,* especially in females brooding late-stage embryos*.* 38 females carried early-stage embryos (I and II) while 52 females carried late-stage embryos (III and IV) under their abdomen. **A** Fecundity, measured in thousands of eggs, in relation to female lobster carapace length (CL), in mm, is significantly lower in female *P. argus* carrying late-stage embryos compared to those carrying early-stage embryos (*n* = 90). Slopes (b) of the relationships between fecundity and lobster CL and coefficient of determination (R^2^) values are displayed for females carrying early and late-stage eggs. **B** Reproductive output (R0), the ratio of the dried female mass to the dried embryo mass, is significantly lower in females carrying late-stage eggs versus females with early-stage eggs (*P* < 0.001). Female *P. argus* mass (g) and *P. argus* entire embryo mass (g) were both log-transformed. Slopes (b) of the relationships between egg mass and lobster mass and coefficients of determination (R^2^) values are displayed for females carrying early and late-stage eggs. The equations are: fecundity (females with early-stage eggs) = -1,100,000 + 17,000(CL), fecundity (females with late-stage eggs) = -430,000 + 9,300(CL), egg mass (females with early-stage eggs) = -1.6 + 1.2(FM), and egg mass (females with late-stage eggs) = -1.5 + 1.1(FM), where CL = lobster carapace length and FM = female mass (dry weight). Female *P. argus* mass (g) and *P. argus* entire embryo mass (g) were both log-transformed

Our initial measure of reproductive performance, fecundity, was lower in females carrying late-stage embryos compared to females carrying early-stage embryos, irrespective of the presence/absence of *C*. *conanobrieni* (Fig. 2A). Average fecundity (± SD; range) for females carrying late-stage embryos was 472,573.2 (± 200,414; 130,054.7 — 913,270.6) embryos lobster^−1^; average fecundity was 631,710.3 (± 251,167.3; 120,319.8 — 1,381,719.5) embryos lobster^−1^ for females carrying early-stage embryos. Fecundity in females carrying early and late-stage eggs, irrespective of egg predator presence or absence, increased with increasing carapace length (R^2^ = 0.53,* P* =  < 0.001). Our analysis of covariance (ANCOVA) detected a statistically significant effect of the two main effects, embryo stage of development (ANCOVA; F = 8.49, d.f. = 1, 86, *P* = 0.005) and lobster carapace length (F = 100.60, d.f. = 1, 86, *P* < 0.001) on fecundity. The interaction term between carapace length and embryo stage of development was also significant (F = 8.64, d.f. = 1, 86, *P* = 0.004), indicating that fecundity increased proportionally more with carapace length in females carrying early-stage eggs than in those brooding late-stage eggs (Fig. 2A).

Like fecundity, reproductive output was lower in females carrying late-stage embryos compared to females brooding early-stage embryos, irrespective of the presence/absence of *C. conanobrieni* (Fig. 2B). Reproductive output ranged between 3.59% and 13.17% with a mean (± SD) of 9.05% (± 1.95), specifically for females carrying early-stage embryos (I and II). Reproductive output for females carrying late-stage eggs (III and IV) varied from 3.24% and 11.47% with an average of 6.97% (± 2.03). The slope of the regression between female lobster dry mass (g) and embryo dry mass (g), after both were log-transformed, exhibited positive allometry (*b* = 1.2, SE_b_ = 0.15); the slope of the line was significantly greater than unity (*t* = 8.30, d.f. = 1, 36, *P* =  < 0.001) meaning that embryo mass grew more than proportionally with a unit increase in female body mass. An ANCOVA found a statistically significant relationship of embryo stage of development (ANCOVA; F = 24.66, d.f. = 1, 86, *P* =  < 0.001) and log-corrected female body mass (F = 110.92, d.f. = 1, 86, *P* =  < 0.001) on log-corrected embryo mass (i.e., reproductive output). The interaction term of the ANCOVA between embryo stage of development and female body mass was not significant (F = 0.15, d.f. = 1, 86, *P* = 0.70) (Fig. 2B).

### The effect of the egg predator on fecundity and reproductive output (RO) of* Panulirus argus*

**Fig. 3:**
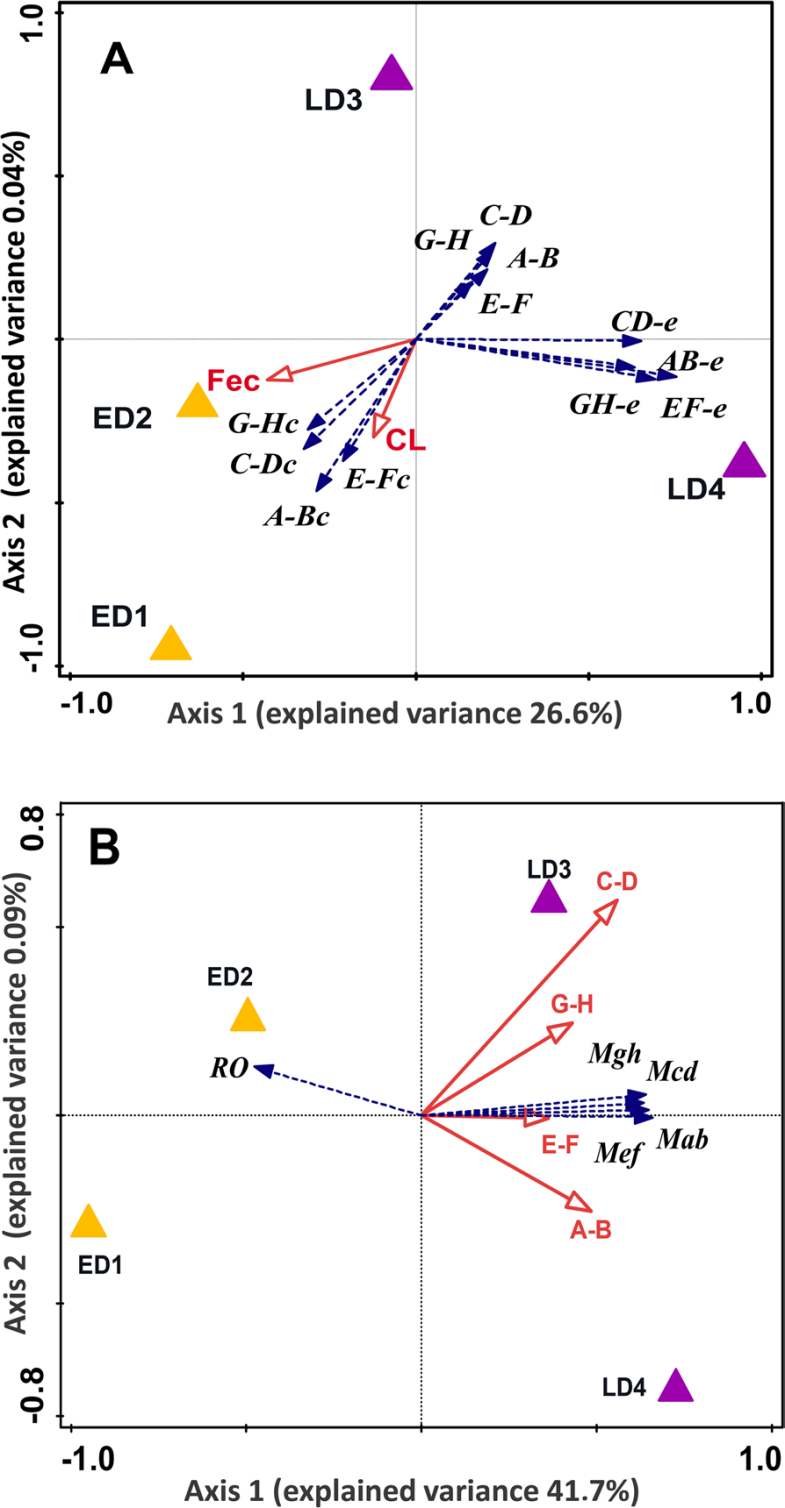
Effects of *C. conanobrieni* adults on fecundity of *P. argus *(a) and on reproductive output and embryo mortality of *P. argus *(b). **A** The abundance of adult *C. conanobrieni* is significantly and negatively correlated with fecundity and reproductive output**.** Redundancy analysis (RDA) biplot showing first and second ordination axes (eigenvalues 0.266 and 0.044, respectively) of adults, cysts and egg abundances of *C. conanobrieni* (blue arrows) with respect to host stages (triangles), host characteristics (red arrows): CL (cephalothorax length) and Fec (fecundity). Length of arrows indicate the direction and rate of increase of the variables, angles between vectors indicate close correlations. **B** The presence of adult *C. conanobrieni* in females carrying late-stage embryos resulted in lower reproductive output and higher embryo mortality compared to females carrying early-stage embryos. Redundancy analysis (RDA) biplot showing first and second ordination axes (eigenvalues 0.417 and 0.009, respectively) of the death that occurred among *P. argus* embryos in the different pleopods (blue arrows) and reproductive output (RO) with respect to abundances of adult *C. conanobrieni* in the different pairs of pleopods (red arrows) and host stages (triangles). A-B: first pair; C-D: second pair; E–F: third pair and G-H: fourth pair. The two graphs show the ordination of predictive and dependent variables. In red arrows, predictive variables: abundance of adult nemerteans per pleopod pair (from anterior to posterior: A-B, C-D, E–F, and G-H), early (ED1, ED2) and late (LD3, LD4) development stages of lobster eggs. In blue arrows, dependent variables: number dead lobster embryos per pleopod pair (Mab; Mcd; Mef; Mgh) and reproductive output (RO) of female lobsters. Each arrow (representing a quantitate variable) points in the direction of the steepest increase of their values. The angle between arrows (red/blue) indicates the correlation between individual predictive variables and/or predictive and dependent variables. The angle between arrows indicates the sign of the correlation between variables. The approximated correlation is positive when the angle is sharp and negative when the angle is greater than 90 degrees. Symbols (triangle) represent levels of a factor, specifically, development stage of lobster eggs (DS). Thus, the distance between these symbols represents the average dissimilarity between the classes (DS) being compared. Closely located symbols and arrows represent an association between levels of a factor and quantitative variables

Our first redundancy analysis (RDA) confirmed that the presence of the egg predator led to losses in host reproductive performance (Fig. 3A). The analysis exploring the relationship between *P. argus* (host) traits and the abundance of different life stages of *C. conanobrieni* resulted in a statistically significant ordination. There was a total explained variance of 31.07% and eigenvalues equal to 0.266 and 0.044 for the first and second ordination axis, respectively (Fig. 3A). Adult worms and their egg masses were significantly more abundant in lobsters brooding late-stage eggs (stages III and IV; both *P* < 0.05) compared to lobsters carrying early-stage eggs (stages I and II) in which adult worms were rarely observed, but nemertean cysts were present. Fecundity was negatively correlated with abundances of adult worms and their eggs present in lobsters carrying late-stages eggs (*P* < 0.05). In addition, a tight association was observed between the abundance of adult worms and worm egg masses located among *P. argus* pleopods. Host body size (CL) was not a statistically significant host trait explaining differences in the abundance of adult worms (*P* > 0.05).

*Carcinonemertes conanobrieni* contributed to declines in reproductive output coupled with significant egg mortality in brooding *P. argus* lobsters. The second RDA resulted in a statistically significant ordination (total explained variance = 41.7%, eigenvalues = 0.4177 and 0.0009 for the first and second ordination axis, respectively; Fig. 3B). Lobsters brooding early-stage eggs (stages I and II) were scarcely infected by adult nemertean worms and egg mortality was significantly lower in these lobsters (*P* = 0.002). Concomitantly, reproductive output was significantly higher in lobsters with early-stage eggs compared to those with late-stage eggs (stages III and IV) in which adult *C. conanobrieni* were abundant and lobster egg mortality was high (*P* = 0.002).

## Discussion

### The effect of *Carcinonemertes conanobrieni* on the reproductive performance of *Panulirus argus*

Limited research has focused on the effect of disease prevalence on reproductive performance in spiny lobsters (see [10] and references therein). This highlights the need for this study on *P. argus* reproductive performance with *C. conanobrieni* detected in ovigerous female brood masses. In the south Caribbean region (Santa Marta, Colombia), *C. conanobrieni* was found in ovigerous *P. argus* females with high prevalence (> 80%), in agreement with [13] and [24] who found a worm prevalence of 87% and 93% in *P. argus* from Saint Kitts, Lesser Antilles and the Florida Keys, USA, respectively. *Carcinonemertes conanobrieni* prevalence estimates from Colombia and previous studies conducted across *P. argus*’ Caribbean distribution have grown from the first report of this worm citing low prevalence at 4.7% [10]. High prevalence of *Carcinonemertes* spp. is common in many crustacean hosts. For instance, *C. errans* prevalence is greater than 90% in the Dungeness crab *Cancer magister* [18] while *C. australiensis* prevalence reaches 60% in *Panulirus cygnus,* a spiny lobster heavily targeted by fisheries in Australia [25]. In addition to *C. conanobrieni* prevalence, the mean intensity of *C. conanobrieni* infection for our study was 11.68 worms lobster^−1^, which was higher than a mean intensity of 5.7 worms lobster^−1^ reported for *P. argus* in Saint Kitts and Nevis, although their mean intensity estimate included the cryptic *Carcinonemertes* sp. which we did not find evidence of in our samples [13]. The prevalence and intensity of *Carcinonemertes* spp., nonetheless, remains to be estimated in most decapod crustacean host species.

Given the high prevalence and mean intensity of *C. conanobrieni* found in *P. argus* brood masses in Colombia, we anticipated the presence of this egg predator to result in significant embryo mortality. We observed that lobsters with *C. conanobrieni* present had embryo mortality that reached up to 48.24%, which coincided with high worm intensity in infected females that averaged 11.68 worms (± 1.98). This was corroborated by both dead and consumed embryos among our subsamples. These estimates of embryo mortality are only slightly lower than *P. argus* in the Florida Keys, whose embryo mortality due to *C. conanobrieni* can reach up to 64.5% in heavily infected females [24]. The lack of embryo mortality in non-infected gravid females coupled with significant embryo mortality in females with adult *C. conanobrieni* present demonstrates the strong negative impact of *C. conanobrieni* on the egg masses of these lobsters. This pattern has been identified in other decapod crustaceans infected with *Carcinonemertes* worms including the Dungeness crab *C. magister*, whose annual embryo mortality exceeded 50% when *C. errans* was present in their brood masses [18]. Additionally, the shore crab *Hemigrapsus oregonensis* experienced 75–100% embryo mortality during outbreak years and 0–52% in non-outbreak periods of *C. epialti* infections [16]. While we did not observe *C. conanobrieni* feeding, direct observations of *C. conanobrieni* consuming *P. argus* embryos has occurred under laboratory conditions [24]. Explicitly, *C. conanobrieni* feeding occurs when the worm punctures a hole in the lobster embryo using its stylet and everts its proboscis to either fully or partially consume the yolk contents using muscular contractions [24]. Additional laboratory studies have confirmed the same suctorial feeding patterns of other *Carcinonemertes* species, including feeding of *C. errans* on *C. magister* embryos [14, 20]. Our results of embryo mortality, reaching almost 50% in some brooding female *P. argus*, provide the first step in aiding future research efforts by establishing baseline embryo mortality estimates for the Colombia *P. argus* fishery due to *C. conanobrieni* infections. Future studies on *P. argus* can aid in determining differences in embryo mortality that occur during *C. conanobrieni* fluctuations i.e. during an outbreak and non-outbreak period in Colombia and other localities in the greater Caribbean basin.

Concomitantly with significant embryo mortality, this study highlights that the presence of *C. conanobrieni* differentially affected the fecundity of female *P. argus*. Our results revealed that infected female *P. argus* carrying late-stage embryos exhibited the lowest fecundity estimates (472,573.2 embryos ± 200,414), which corresponded with having the highest abundance of worms present in their brood masses. Of these worms found in late-stage brooding females, the majority were adult *C. conanobrieni,* either free roaming or in mucus sheaths, in addition to nemertean egg masses intertwined within *P. argus* embryos. Contrarily, females carrying early-stage embryos only had worm cysts present in their embryo masses. This suggests that there is a significant, disproportionate negative effect of adult *C. conanobrieni* on female fecundity throughout lobster embryo development.

Parallel to female lobster fecundity, reproductive output was also negatively affected by the presence of *C. conanobrieni* in lobster brood masses. Specifically, reproductive output estimates were higher for females carrying early-stage embryos compared to females carrying late-stage embryos. Our second RDA confirms a significant difference in reproductive output between females carrying early vs. late-stage eggs, which coincided with higher adult worm abundance and higher egg mortality in the brood mass of females carrying late-stage embryos. Declines in reproductive output between the different embryo stages described here are in line with previous limited information suggesting declines in reproductive output of infected *P. argus* females in the Florida Keys population [10], highlighting the increased intensity of infection that ensues as lobster embryos progress from early to late stages of development. To the best of our knowledge, reproductive output has not been estimated in other spiny lobster species [see [25–54]. However, reproductive output, as estimated in this study, has been widely reported for other decapod crustaceans [54]. On the west coast of the USA, *C. magister* also experienced losses in reproductive output with increasing *C. errans* prevalence as hosts became gravid [18, 20, 46]. The mechanism behind the progression of *C. conanobrieni* infection throughout embryonic development remains to be addressed, however, this may indicate that the life cycle of *C. conanobrieni* is closely linked to the progression of *P. argus* embryo development and subsequent hatching. Supplementing this, *P. argus* females in Saint Kitts and Nevis had a high presence of an undescribed *Carcinonemertes* sp. in their gills during non-peak periods of reproductive activity [47] and *C. errans* infests the brood mass of the Dungeness crab *C. magister* by living on the exoskeleton until 1–2 days after host oviposition [18].

The reproductive performance estimates described in this study can add to the growing literature of *Carcinonemertes* spp. impacts with the discovery of new, previously uninfected hosts. Previous research has compared the occurrence of nemertean worms in the genus *Carcinonemertes* across exploited decapod crustaceans and their variability in space and temporally [15]. Conclusions as to whether fishing pressure promotes infection by *Carcinonemertes* spp. and subsequent outbreaks has yet to be determined, however, the importance of monitoring populations becomes key to determining if infection by these worms is temporary or ongoing [15]. This study also highlights the importance of using methods other than the most used, brood mortality, to investigate the negative consequences of infection on host reproductive performance. This is warranted because re-occurring brood loss may lead to host female fitness being dramatically reduced during infection. In corroboration with this study and others focused on *P. argus* reproductive performance [10], researchers have concluded that wild lobster stocks under repeated infestation by *Carcinonemertes* worms may be in decline due to a substantial loss in the number of larvae released into the fishery [13].

## Conclusion

Our study provides the first estimates of embryo mortality and reproductive performance of female *P. argus* infected with *C. conanobrieni* in a southern Caribbean (Colombia) population. This egg predator’s initial discovery in the Florida Keys has led to more studies reporting the prevalence of *C. conanobrieni* across *P. argus*’ distribution in the greater Caribbean, Saint Kitts and Nevis, and Colombia [9, 12, 13, 24]. Our results suggest that the high prevalence of this egg predator is causing a decline in individual-level reproductive performance measures. Multiple researchers have justified for the temporal monitoring of this population and other populations across the entire Caribbean given the growing prevalence and impacts of *C. conanobrieni* and undescribed *Carcinonemertes* sp. on *P. argus* [12, 13]. In agreeance with other researchers, we encourage the sampling of *P. argus* reproductive performance and overall health across its other Caribbean localities including Belize, Bermuda, and the Cayman Islands, all of which have also not yet been investigated for *C. conanobrieni* presence. Additionally, since *Carcinonemertes* sp. can also be found to infect multiple hosts, we suggest the screening of other exploited and non-exploited decapod crustaceans, including the spotted spiny lobster *P. guttatus,* the smoothtail spiny lobster *P. laevicauda,* and Florida stone crabs *Menippe* spp.

## Methods

### Sampling of *Panulirus argus*

Sampling was conducted with the authorization of the “Autoridad Nacional de Licencias Ambientales ANLA'' under permit 1293—2013 “Permiso marco de recolección de especímenes de especies silvestres de la diversidad biológica con fines de investigación científica no comercial, resolución” given to Universidad del Magdalena, Santa Marta, Colombia. Between March 16^th^ and October 26^th^, 2019, a total of ninety *P. argus* ovigerous females were caught by artisan fishers from shallow hard-bottom environments north of the Gulf of Salamanca (latitude: 11.03 to 11.06 ºN, longitude: -74.42 to -74.62 ºW), Magdalena, Colombia. Immediately after capture, lobsters were transported in ice-chests to the laboratory of the “Grupo de Investigación en Manejo y Conservación de Flora Fauna y Ecosistemas Estratégicos Netropicales MIKU'' at Universidad del Magdalena and euthanized within 24 h. We note that lobsters were not individually maintained in the ice-chests. Preliminary observations have shown that *C. conanobrieni* worms do not move much, are not efficient at crawling, and do not swim (AB, NS, and JAB). Therefore, the probability of worms moving among lobsters during their transportation to the laboratory was null.

### *Carcinonemertes conanobrieni* prevalence, intensity and embryo mortality

To understand the effect of *C. conanobrieni* on *P. argus* reproductive performance and embryo mortality, we measured prevalence and intensity of the egg predator on individual females. In the laboratory, each brooding lobster was measured (CL = length of the cephalothorax expressed in mm, precisio*n* = 0.1 mm) and the developmental stage of their brooded embryos were classified following [10] as: stage I, embryos with evenly distributed yolk, no separation between yolk and chorion; stage II, embryos with cellular differentiation, yolk and chorion begin to separate; stage III, embryos with pigmentation in the eyes; stage IV, embryos with developed eyes, thoracic appendages, and chromatophores (red pigments). Next, all eight pleopods (each carrying eggs) were carefully dissected with fine tip forceps. A random subsample of ~ 1000 embryos (~ 2–3 mm^3^) was isolated from each pleopod egg mass (*n* = 8 per brooding female) and transferred to Petri dishes containing micro-filtered seawater. Each sub-sample was then inspected for the abundance (if worms were observed) and presence or absence of the different life stages of *C. conanobrieni* under a dissecting microscope (Stemi DV4, Carl Zeiss, Germany). The different ontogenetic stages of *C. conanobrieni* included (i) adults (either roaming among the lobster eggs or in sheaths), (ii) encysted juveniles, and (iii) embryo aggregation (worm egg masses) [10, 24]. Additionally, we determined the presence or absence of dead lobster embryos and totaled dead embryos for each lobster egg sub-sample. Dead lobster embryos were recognized as fully or partially empty cases or as cases with lobster embryos having abnormal size (smaller or larger than the surrounding embryos), shape (usually a-symmetrical), and coloration (either a dark brown or a light, milky orange) [10].

Egg predator prevalence was estimated as the number of infected lobsters with at least one *C. conanobrieni* in at least one pleopod divided by the total number of lobsters sampled (*n* = 90). *C. conanobrieni* intensity was calculated as the average number of egg predators found per examined pleopod (*n* = 8) in each lobster [48]. Finally, egg mortality was expressed as the average proportion of dead eggs observed per pleopod in the 1000 embryos subsamples.

### Lobster fecundity and reproductive performance

We calculated two individual-level reproductive performance parameters in infected and uninfected *P. argus* brooding females: fecundity and reproductive output (RO). In this study, fecundity is defined as the total number of embryos carried underneath the abdomen by a brooding female lobster and the words eggs or embryos are used indistinctly. Reproductive output refers to the amount of biomass or fraction of energy that the female invests in reproduction, and this parameter was estimated as the ratio between the dry weight of the embryos and the dry weight of the lobster body [10, 49].

Our two measures of reproductive performance, fecundity and reproductive output, were evaluated following past investigations into ovigerous female *P. argus* [10]. Five random subsamples of 100 embryos each, the remaining embryonic mass (MR), and the whole body of each lobster, were dried separately at 68 °C until constant weight (approx.120 h) and then weighed with an analytical balance (OHAUS; 0.1 mg). Fecundity was calculated with the formula: F = [(((Mass_embryos_ / Average (Mass_sub1_, Mass_sub2_, Mass_sub3_, Mass_sub4_, Mass_sub5_))*100) + 500)]; where F = the total number of embryos, Mass_embryos_ = the dry weight of the remaining embryo mass after the five 100 subsamples were removed, Mass_sub#_ = the dry weight of one of the embryo subsamples of 100, and the 500 added back in represents the total number of embryos removed for the sub-samples [10]. Reproductive output was calculated with the formula: RO = Mass_embryos_ / Mass_female_; where RO = reproductive output, Mass_embryos_ = the dry weight of the five 100 eggs sub-samples plus the remaining embryo mass after the subsamples were removed, and Mass_female_ = the dry weight of the female lobster after their eggs were extracted.

### The effect of the egg predator on fecundity and reproductive performance of* Panulirus argus*

We used Redundancy Analysis (RDA), a multivariate constrained ordination technique [50–52], to (i) determine which host (lobster) traits were the most significant to explain variation in the occurrence of different life stages of *C. conanobrieni* in lobster egg masses and (ii) examine the effects of *C. conanobrieni* (adults) on lobster egg mortality and fecundity/reproductive output. RDA extracts and summarizes the variance in a set of dependent variables (matrix Y) that can be explained by a set of (quantitative or qualitative) predictive variables [50, 51]. In the RDA analysis, multiple linear regressions are used to ‘explain’ variation between the independent and dependent variables, and these calculations are performed within an iterative procedure to find the best ordination of the samples [53]. Graphically, the results of a RDA are presented in the form of biplots showing response variables, qualitative explanatory variables as centroids, and quantitative variables as vectors. RDAs were performed in CANOCO 4.5 for Windows using the automatic forward selection procedure, and the statistical significance of each predictor variable was determined using 500 Monte Carlo permutations. Differences at alpha < 0.05 were considered statistically significant.

In the first RDA, we analyzed the relationship between host traits (X-matrix) and the occurrence of different nemertean life stages per pleopod (Y-matrix). The explanatory matrix in this first analysis included log-transformed values of carapace length (LC), dry body weight (dBW), dry brood weight (DBW), fecundity (n° eggs × 10^3^), and host egg stage (*n* = 4 stages). The response matrix included n° adult nemerteans, n° nemertean cysts, and n° egg sacs recorded in each of the four pairs of pleopods. Prior to the analysis, we explored collinearity between different predictor variables and found high collinearity (assessed using variance inflation factors [VIFs]) between dBW and DBW, dBW and fecundity, and DBW and fecundity (each observed VIF´s > 30). Therefore, dBW and DBW were excluded from the analysis to avoid model over-parameterization.

In the second RDA, we examined the effects of *C. conanobrieni* on lobster egg mortality and reproductive output. The predictor matrix in this analysis included the abundance of adult nemertean worms in each one of the four pairs of pleopods as well as host development stage while the dependent Y- matrix consisted of host-dead embryos (log[x + 1] transformed data) recorded in each of the four pairs of pleopods and reproductive output. No evidence of collinearity between the predictor variables was found prior to the analysis (VIF < 2.9).

## Supplementary Information

**Additional file 1:** **Supplementary Table 1.** First RDA constrained analysis of simple effect terms. **Supplementary Table 2.** Second RDA constrained analysis simple effect terms.

## Data Availability

All datasets on which the conclusions of the manuscript rely are presented in the main text of the manuscript.

## Abbreviations

CL: Carapace length
SD: Standard deviation
ANCOVA: Analysis of covariance
RO: Reproductive output
RDA: Redundancy analysis
min: Minimum
max: Maximum
dBW: Dry body weight
DBW: Dry brood weight
Fec: Fecundity
VIFs: Variance inflation factors
