# Supplementary material for: The effect of the egg-predator Carcinonemertes conanobrieni on the reproductive performance of the Caribbean spiny lobster Panulirus argus
Source: BMC Zool. 2023 Jun 26;8:6. doi: 10.1186/s40850-023-00165-w (PMC10291781; doi:10.1186/s40850-023-00165-w)

Supplementary Table 1. First RDA constrained analysis of simple effect terms.

Table S1. Results of RDA analyses showing simple terms effects of host predictive variables on nemertean abundances. Explain% = variance explained by the predictive variable; pseudo-F, probability (P), and Bonferroni adjusted probability (Padj).


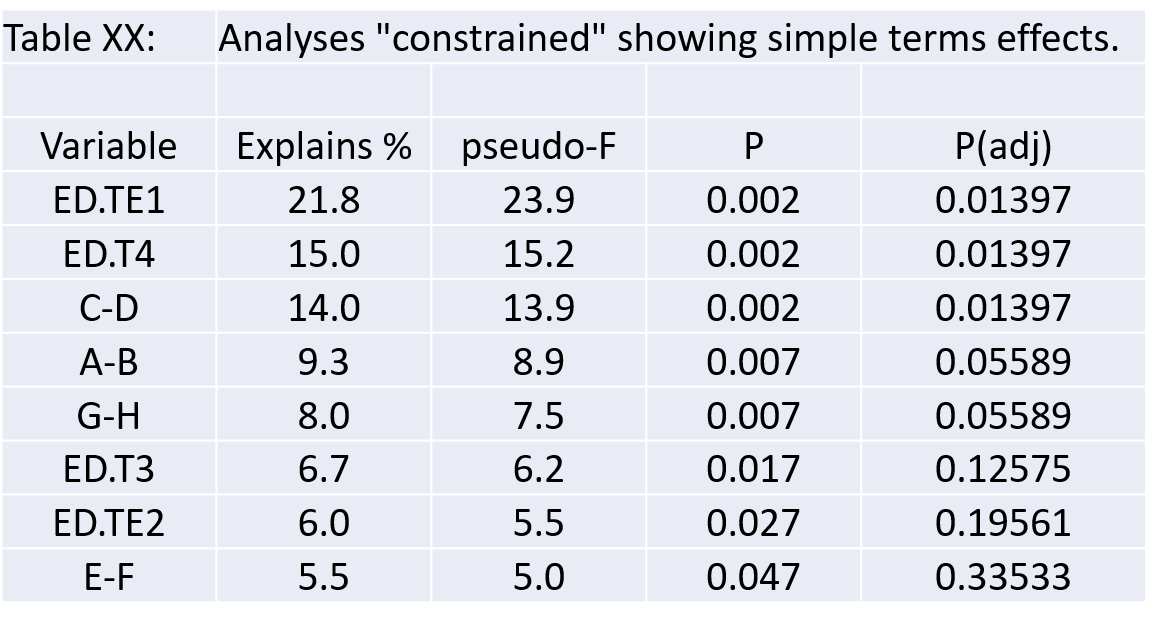


Supplementary Table 2. Second RDA constrained analysis simple effect terms.

Table S2. Results of RDA analysis showing simple terms effects of host predictive variables on nemertean abundances. Explain% = variance explained by the predictive variable; pseudo-F, probability (P), and Bonferroni adjusted probability (Padj)


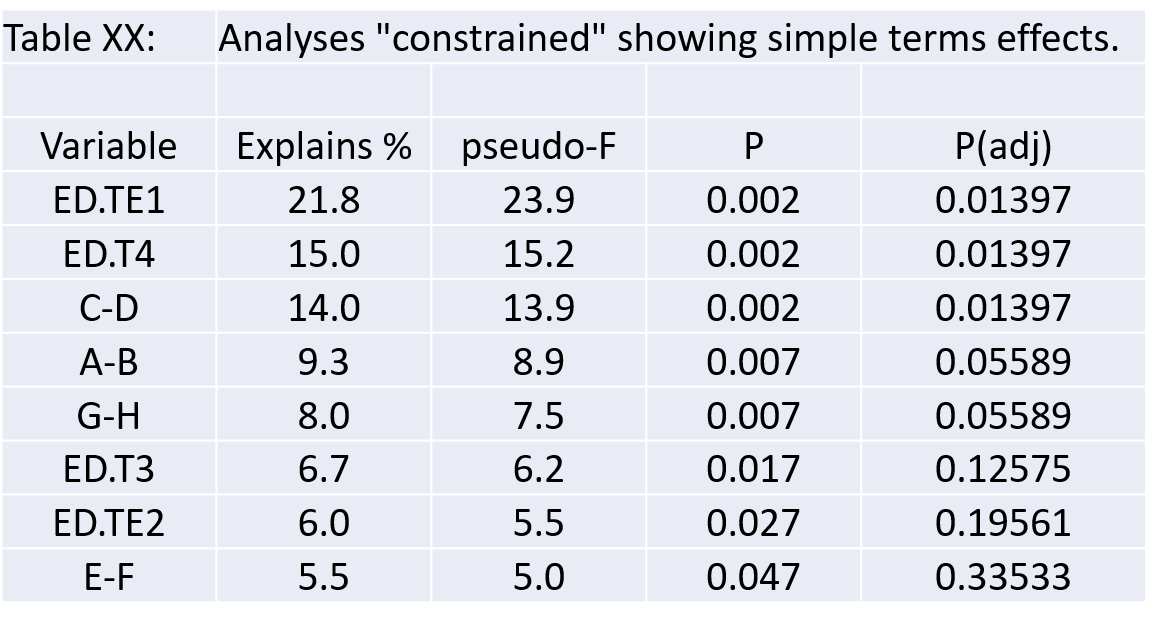

Supplement: Supplementary file 1 — Additional file 1: Supplementary Table 1. First RDA constrained analysis of simple effect terms. Supplementary Table 2. Second RDA constrained analysis simple effect terms. [file 40850_2023_165_MOESM1_ESM.docx]
